# Editorial: The Deadly Secrets of *C. Difficile*—Insights Into Host-Pathogen Interaction, Volume II

**DOI:** 10.3389/fmicb.2022.896979

**Published:** 2022-04-06

**Authors:** Meina Neumann-Schaal, Uwe Groß, Ingo Just, Dieter Jahn

**Affiliations:** ^1^Research Group Metabolomics, Leibniz-Institute DSMZ–German Collection of Microorganisms and Cell Cultures, Braunschweig, Germany; ^2^Braunschweig Integrated Centre of Systems Biology (BRICS), Braunschweig, Germany; ^3^Institute of Medical Microbiology and Virology, University Medical Center Göttingen, Göttingen, Germany; ^4^Institute of Toxicology, Hannover Medical School, Hannover, Germany; ^5^Institute of Microbiology, Technische Universität Braunschweig, Braunschweig, Germany

**Keywords:** *Clostridioides* (*Clostridium*) *difficile*, *Clostridioides* (*Clostridium*) *difficile* infection, host pathogen interaction, toxins A and B *Clostridium difficile*, binary toxin CDT

## Introduction

*Clostridioides* (formerly *Clostridium*) *difficile* is an anaerobic, spore-forming bacterium, widely distributed in soil, water, animals, and the gut of healthy humans (Hall and O'Toole, 1935; al Saif and Brazier, 1996; Lawson et al., 2016). The symptoms of *C. difficile* infection (CDI) range from relatively mild diarrhea to severe life-threatening pseudomembranous colitis, toxic megacolon with bowel perforation and a concomitant sepsis (Lessa et al., 2015). In the last two decades, an emerging number of nosocomial and community-acquired infections was reported worldwide (Bartlett, 2006; Rupnik et al., 2009; Lessa et al., 2015). CDI is commonly, but not necessarily associated with previous administration of antibiotics. Further risk factors are age, cancer treatment and immunosuppression. However, CDI also affects individuals without these classical risk factors (Bignardi, 1998; Rupnik et al., 2009).

## The Clostridial Toxins and Beyond

*C. difficile* harbors different secreted and surface proteins responsible for the colonization of the colon and a subsequent inflammatory reaction in *C. difficile* infection. The most important and best characterized ones are three large clostridial toxins: toxin A and toxin B and in some bacterial strains, the binary toxin CDT.

In an approach by Petersen et al., toxin-catalyzed GTPase glucosylation resulted in subsequent cell cycle arrest which on the other hand was causative for decreased repair capacity of the colonic epithelium and delayed epithelial renewal. The role of toxin A and toxin B in treatment of *C. difficile* infection is elucidated in a study by Papatheodorou et al. to understand how important cholesterol is for the translocations process of the toxins into the cytoplasm. Two further publications focus on the role of the binary toxin CDT. In a study by Marquardt et al., binary toxin-induced activation of the innate immune system (MAIT cells) was identified to be partially MR1-dependent and allow hypervirulent *C. difficile* to overcome cellular barriers. Proteomic analysis of the cellular effects of the binary toxin on human cells by Stieglitz et al., showed only moderate changes on the overall proteome level but strong changes in the phosphorylation level. CSNK2A1 might act as an effector kinase in response to CDT.

In a review by Nibbering et al., both, current knowledge on toxin and non-toxin based host immune response are summarized and compared. The authors highlight that future research should focus on the immune responses to non-toxin proteins and non-toxigenic strains as starting point for non-antibiotic therapeutic approaches.

## Physiology and Host Response

In the host, *C. difficile* is exposed to the host immune response and multiple stress factors. With the increasing knowledge on the pathogen itself, multifactorial systems in the human host get into focus. Biofilms are discussed as a form of persistence for *C. difficile* in the human host. Brauer et al. used a proteomics approach for understanding the biofilm formation and the role of proteins on the cell surface and the underlying regulatory network. Motility as one of the key patterns in biofilm formation is addressed in two further publications.

The regulation of flagellin synthesis was studied by Zhu et al. showing that the carbon storage regulator A modulates flagellin expression along with indirect effects of the flagellin assembly factor FliW. Schwanbeck et al. studied the swimming behavior in depending on environmental parameters, specifically also addressing the viscoelastic properties of the environment. Three further articles focus on different aspects of the host microbiome and the influence on *C. difficile*. In a review on recent developments, Vasilescu et al. focus on the microbiota profiles in infants and adults colonized or infected with *C. difficile* and provide insights into the role of protective microbial species. The impact of bile acids on *C. difficile* was studied by Metzendorf et al. and showed the effects on flagella, toxins and specifically the membrane composition. A study by Aguilar-Zamora et al. with isolates from patients in Mexico showed a high share of multidrug resistant strains and the importance of whole genome sequencing.

## Conclusion and Outlook

The research on *C. difficile* of the last years gives in fact deeper insights into the process of pathogenesis but also increases complexity. Complexity is further increased by the diversity of strains. The simple story of two villains—toxins A and B—is thing of the past. The studies of this volume contribute to the idea that secreted proteins/toxins and adhesion proteins are in addition to the toxins modulators of the host response. CDT seems to affect the immune system rather to act as cytotoxin. Persistence of *C. difficil*e is no longer more simply based on sporulation but biofilm formation contributes as well. The gut microbiome not only interacts with *C. difficile*—outgrowth and sporulation—but also with colonic mucosa and the gut immune system. All these interdependencies define the gut environment to be protective or permissive for inflammation and the clinical outcome. Thus, the understanding of the effects of atoxinogenic, that means asymptomatic *C. difficile* strains on colonic mucosa and the immune system evolves to be the foundation of the development of non-antibiotic treatment of CDI.

## Author Contributions

All authors listed have made a substantial, direct, and intellectual contribution to the work and approved it for publication.

## Conflict of Interest

The authors declare that the research was conducted in the absence of any commercial or financial relationships that could be construed as a potential conflict of interest.

## Publisher's Note

All claims expressed in this article are solely those of the authors and do not necessarily represent those of their affiliated organizations, or those of the publisher, the editors and the reviewers. Any product that may be evaluated in this article, or claim that may be made by its manufacturer, is not guaranteed or endorsed by the publisher.

## References

[B1] al Saif N. Brazier J. S. (1996). The distribution of *Clostridium difficile* in the environment of South Wales. J. Med. Microbiol. 45, 133–137. 10.1099/00222615-45-2-133 8683549

[B2] Bartlett J. G. (2006). Narrative review: the new epidemic of *Clostridium difficile*–associated enteric disease. Ann. Intern. Med. 145, 758–764. 10.7326/0003-4819-145-10-200611210-00008 17116920

[B3] Bignardi G. E. (1998). Risk factors for *Clostridium difficile* infection. J. Hosp. Infect. 40, 1–15. 10.1016/S0195-6701(98)90019-6 9777516

[B4] Hall I. C. O'Toole E. (1935). Intestinal flora in new-born infants: with a description of a new pathogenic anaerobe, *Bacillus difficilis*. Am. J. Dis. Child 49:390. 10.1001/archpedi.1935.0197002010501025996397

[B5] Lawson P. A. Citron D. M. Tyrrell K. L. Finegold S. M. (2016). Reclassification of *Clostridium difficile* as *Clostridioides difficile* (Hall and O'Toole 1935) Prevot 1938. Anaerobe 40, 95–99. 10.1016/j.anaerobe.2016.06.008 27370902

[B6] Lessa F. C. Mu Y. Bamberg W. M. Beldavs Z. G. Dumyati G. K. Dunn J. R.. (2015). Burden of *Clostridium difficile* Infection in the United States. N. Engl. J. Med. 372, 825–834. 10.1056/NEJMoa1408913 25714160 PMC10966662

[B7] Rupnik M. Wilcox M. H. Gerding D. N. (2009). *Clostridium difficile* infection. Nat. Rev. Microbiol. 7, 526–536. 10.1038/nrmicro2164 19528959

